# An Analysis of Railway Activity Using Distributed Optical Fiber Acoustic Sensing

**DOI:** 10.3390/s25134180

**Published:** 2025-07-04

**Authors:** Thurian Le Du, Arthur Hartog, Graeme Hilton, Roman Didelet

**Affiliations:** FOSINA, 23 rue du port, 92000 Nanterre, France; arthur.hartog@fosina.fr (A.H.); ghilton@fosina.fr (G.H.); rdidelet@fosina.fr (R.D.)

**Keywords:** fiber, railway, DAS

## Abstract

Distributed acoustic sensing (DAS) is a highly effective method of monitoring all kinds of intrusions on railway tracks. These intrusions represent a real problem in the railway sector, as they can lead to human deaths or damage to railway tracks, and these intrusions may be human or animal. A fiber was deployed along 12 km of track in a railway test center, enabling us to acquire data day and night. A data acquisition campaign was carried out in April 2023 to capture the signatures of several scenarios (walking, digging, falling rocks, etc.) in order to train machine learning models and prevent any intrusion by detecting and classify these intrusion. The study shows the diversity of signals that fiber can acquire in the rail sector and the machine learning model performance. Signals associated with the presence of animals are also presented.

## 1. Introduction

The imperative to ensure the safety and efficiency of railway operations has catalyzed the development of advanced monitoring technologies. Among these, fiber optic sensors have emerged as a promising solution for real-time, large-scale infrastructure surveillance. Fiber optic sensors offer several advantages: they can detect minute variations, crucial for early failure detection; they are immune to electromagnetic interference; and they can be deployed in harsh environment. Finally, a single sensor can monitor extensive distances, reducing installation and maintenance costs unlike more conventional sensors, which are generally positioned in a single location.

Railway companies have a critical need for the rapid detection of animal and human intrusions on railway tracks. These intrusions pose significant risks, including collisions with animals or people which can cause severe injuries or fatalities, damage to trains, and derailments. Thanks to the data collected using the fiber, machine learning can be used to detect and classify these intrusions and alert the relevant authorities.

Many recent studies have focused on the use of machine learning for intrusion detection [1,2], track detection [3], and train integrity monitoring [4,5,6]. Gan et al. [7] have shown that the use of FBG (Fiber Bragg Grating) can be relevant in certain cases, but FBG sensors are better suited for applications requiring precise and localized measurements of strain and temperature. DAS (Distributed Acoustic Sensors) can monitor very long distances, often up to 100 km or more, with a single optical fiber. This allows for extensive coverage with minimal infrastructure. This technology is particularly sensitive to vibrations and acoustic waves, making them ideal for detecting intrusions, vehicle movements, or suspicious activities along railway tracks. Several studies have shown good results in detecting and classifying intrusions, but these intrusions are only of an anthropogenic nature [8,9,10]. There have been a few reports of animals being detected, but these are domestic animals (horses, sheep flock) that present little risk of trespassing on railway lines [11]. Elephant detection is also a subject of interest, particularly in India [12], but to date, there have been no published results with fiber optic sensors on this subject.

Another concern is monitoring faults on the railway line. Recent studies have shown the usefulness of fiber optic sensors for detecting faults on the track [13,14,15,16]. Structural health monitoring is a highly studied area in the railway sector, but there are relatively few studies showing actual cases of intrusions on railway lines. In this paper, we propose taking stock of the various signatures acquired during the measurement campaign. For the rest of the article, readers will find a list of abbreviations at the end.

Section 1 presents the physical principles of fiber optic sensors, while Section 2 describes the various players involved and the type of data and parameters used. Finally, Section 4 presents the results of this measurement campaign including animal intrusions.

## 2. Materials and Method

This section describes the field of distributed fiber measurement, the test environment, the sensors used, and the associated acquisition parameters.

### 2.1. Distributed Fiber Optic Sensing

Distributed Fiber Optic Sensing (DFOS) is the technology that underpins the work described in this article.

Optical fiber sensors (OFS) are devices that use an optical fiber to sense a measurand, such as temperature, strain, pressure, vibration, or current. In general, they consist of an interrogator—an opto-electronic unit that launches probe light into the fiber and that analyses the light transmitted by, or reflected from, the fiber. At the sensing location, the light is modulated by the measurand through at least one of its attributes, such as amplitude, phase, polarization, group delay, coherence, or frequency. By analyzing the light exiting the fiber, the value of the measurand can be determined.

Distributed fiber optic sensors [17] are a special class of OFS that provide the spatial distribution of the measurand along the fiber. Although many types of DFOS exist, they are, in general, based on optical time-domain reflectometry, in which a pulse is launched into the sensing fiber. As this probe pulse travels down the fiber, it scatters light in all directions, and some of that scattered light is re-captured by the fiber in the return direction. The time at which the returning light is measured is, therefore, directly related to the location from which it was scattered. For completeness, it should be noted that similar results can be achieved with other modulation formats, such as frequency-modulated, continuous wave, or pseudo-random encoding.

In DFOS, the sensitivity to specific measurands is achieved by analysis of the spectrum of scattered light. For example, the intensity of the Raman bands is commonly used for temperature sensing [18], and the Brillouin lines provide information on both temperature and strain [19]. In this paper, we use the phase of the Rayleigh backscatter to sense changes in the strain of the fiber with sensitivity in the pε range.

In contrast to Raman and Brillouin, which both shift the frequency of the scattered light and result in signals that are incoherent with the probe light, Rayleigh scattering returns light at the same frequency as the probe light and with a stable-phase relationship to it.

In our work, the interrogation is based on optical time-domain coherent Rayleigh reflectometry [17], in which pulses of highly coherent light are launched into the fiber. The phase of the backscattered light is detected and differentiated across a section of fiber known as the gauge length (GL). The differentiated phase is tracked over time for each successive probe signal, for each resolved fiber location. Changes in the differential phase across the GL is directly related to changes in the physical length of the GL, and so this approach allows us to detect and quantify minute strains at each location along the fiber. A change in phase δϕ at location z is directly related to the change δl in the dimensions of the GL through

(1) $$\delta \Phi \left(z\right)=\frac{4\pi }{\lambda }\;n\cdot \delta l\left(z\right)\cdot \xi$$

where λ is the source wavelength, n is the refractive index of the fiber, and ξ is a coefficient that corrects the change in the physical length of the GL to take into account the strain-optical effect, i.e., the fact that when the fiber is strained, its refractive index is altered. In our conditions, ξ = 0.79 [17].

This phase comparison is achieved with each repetition of the probe signal, and so a picture of the dynamic strain is obtained for each location along the fiber. The rate at which the fiber is probed depends on the length of the fiber, but it can easily exceed 1 kHz, which is more than sufficient for analyzing the vibrations caused by the intrusion events that we describe in the following sections.

### 2.2. Field Trial Setup

#### 2.2.1. Fosina

FOSINA (Fiber-Optic Sensing with Intelligent Native Architecture) is a young start-up specializing in fiber optic sensors, and it provides a solution, known as DxS, that can combine distributed acoustic sensing (DAS), distributed temperature sensing (DTS), distributed strain sensing (DSS), and distributed temperature gradient sensing (DTGS) in a single 4U interrogator [20].

#### 2.2.2. CEF

The Centre d’Essais Ferroviaires (CEF) was set up in 1998 at the instigation of the main players in the rail industry. It is a unique facility in France, whose purpose is to hire out its infrastructure and services for railway testing, maintenance, and training. Initially designed for the development of new rolling stock and signaling systems, from urban to conventional, it can also be used to test infrastructure elements (track or catenary components). The center has a line almost 12 km long, capable of 160 km/h, electrified at 25 kV and 1500 V, as well as areas equipped for shunting tests.

**Figure 1:**
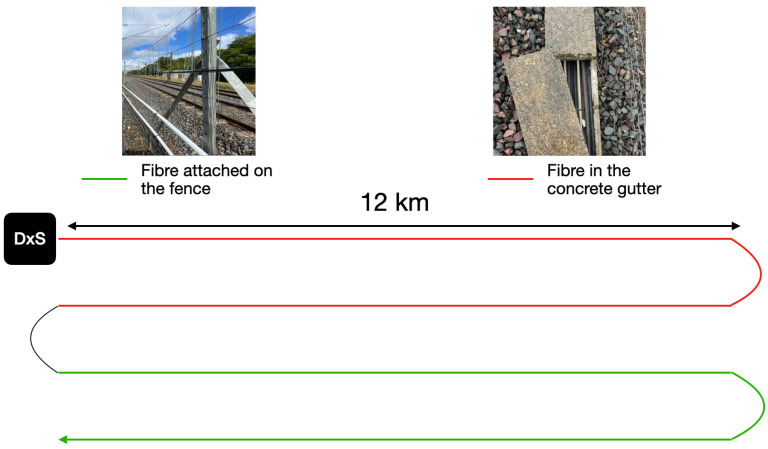
Diagram showing fiber layout.

The fiber runs over 12 km of a track four times, with a return trip in the concrete gutter and another return trip attached to the fence, as shown in Figure 1. In this study, we are interested in the data acquired by the portion of fiber in the concrete gutter. The fiber used is a single-mode fiber.

#### 2.2.3. DxS Acquisition Parameters Used

Given that the total length of the fiber is approximately 46 km, the acquisition sampling frequency is 500 Hz. The gauge length applied is 5.7 m, and the spatial resolution (the separation between successive points in the processed output of the interrogator) is approximately 1 m as shown in Table 1. The phase of the disturbances detected by the fiber from the backscattered signal was collected on a daily basis. The FBE (frequency band energy), the acoustic energy measured at each location in a number of pre-defined spectral intervals, was also determined.

## 3. Results

In this section, the signals captured are presented, as is the work involved in detecting events using machine learning.

### 3.1. Data from the Acquisition Campaign

**Figure 2:**
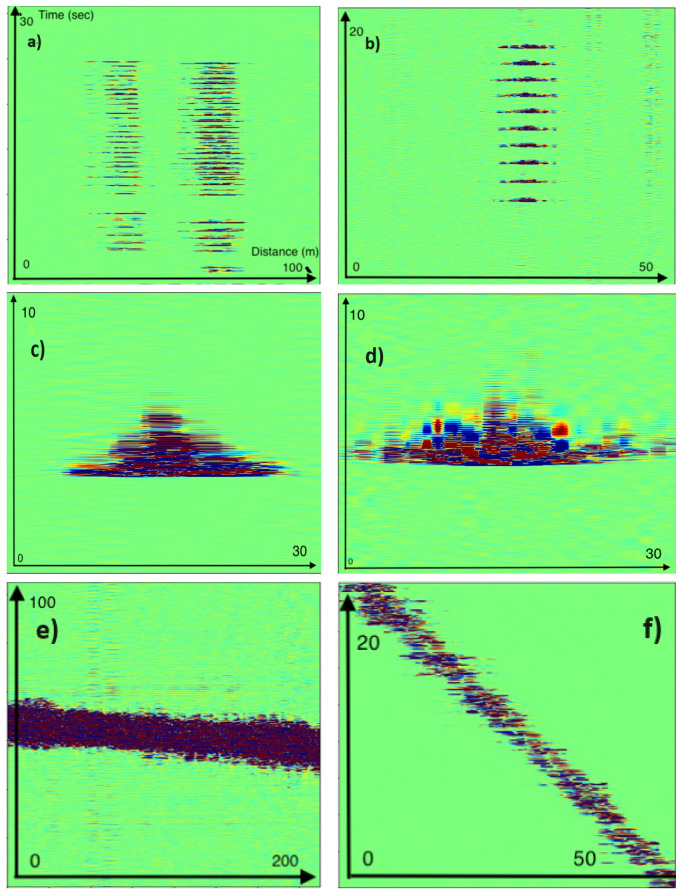
Sample of phase data from several activities captured during the campaign. (**a**) Digging; (**b**) hammer blows on the railway; (**c**) rock drop, rolling down the slope on the opposite duct; (**d**) dropping a rock on the railway line; (**e**) train; (**f**) walking.

Several activities were carried out during the campaign to train our machine learning models. The data was acquired with a sampling frequency of 500 Hz and a gauge length of 5.7 m. Figure 2 shows a sample of activities carried out during the campaign.

We note that each activity has its own signature. Activity (a) consisted of digging up the ballast in the middle of the track at two different locations simultaneously. Activity (b) consisted of hammering the track at regular intervals. (c) is a rock falling down to the track, (d) is a rock falling directly onto the ballast, (e) is a train passing by, and (f) is a person walking on the duct. Other activities included climbing the fence and cutting a wire that had been attached to the fence has been captured.

**Figure 3:**
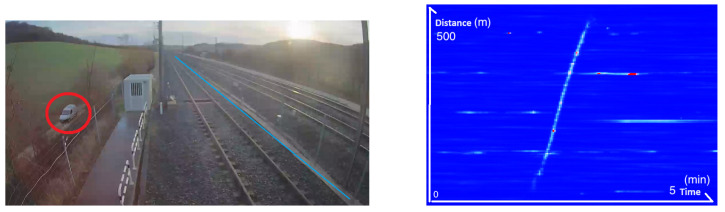
**Left**: Image taken from a CCTV camera showing the car driving past the railway line. The car is surrounded by a red circle, and the location of the fiber is indicated by a blue line. **Right**: The associated frequency band energy between 0 and 250 Hz showing the movement of the car.

These activities were carried out close to the fiber, but several events were measured at locations further away from the fiber. For example, we were able to measure the passage of a car on a road that runs alongside the railway at a distance of 15 m from the fiber as shown in Figure 3.

**Figure 4:**
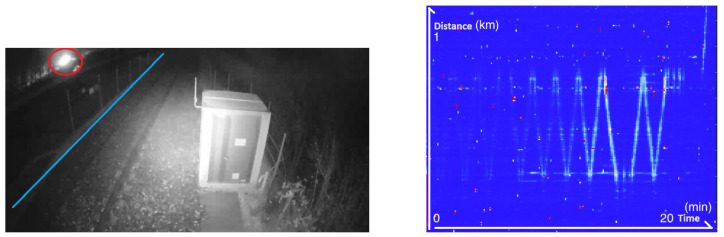
**Left**: Image taken from a CCTV camera showing the light generated by the farmer during the night. The light is surrounded by a red circle and the location of the fiber is indicated by a blue line. **Right**: The associated frequency band energy between 0 and 250 Hz showing the movement of the farmer machine.

We can also detect the farmer working in their field next to the track. In this example, we can see the energy increase as the farmer gets closer to the railway track, moving back and forth, as shown in Figure 4. The nearest edge of the field is around 30 m away from the fiber.

**Figure 5:**
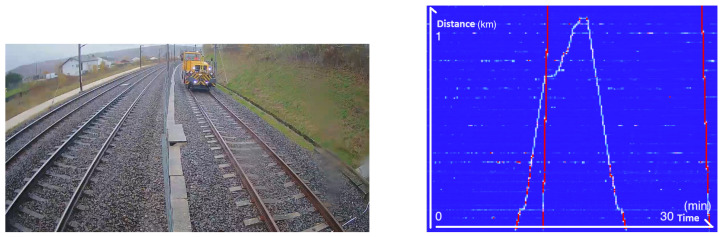
**Left**: Image taken from a CCTV camera showing the machine. **Right**: The associated frequency band energy between 0 and 250 Hz showing the round trip of the machine.

The test center carries out regular maintenance operations along the DTT (Dynamic Test Track). To do this, they move slowly with machines. As shown in Figure 5, we know exactly where the machine is at all times. In this example, the machine makes a round trip. The two near vertical red lines are trains running on the tracks on the other side of the fence.

**Figure 6:**
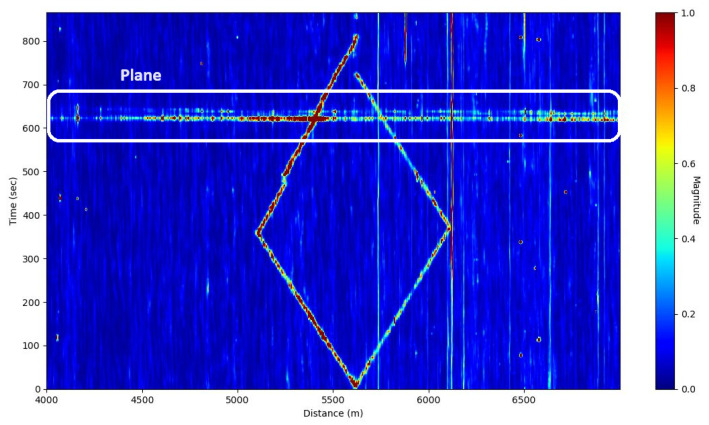
Frequency band energy showing the walking test with the plane’s passage (white rectangle).

Finally, a walking test was carried out at a distance of 5 m from the fiber. Two people walked about 500 m in opposite directions and then retraced their steps. We can see that the cable sensitivity can vary with distance. At approximately 620 s, a military aircraft flew over the railway line. The sound generated by the aircraft was measured by the fiber as shown in Figure 6. We can note that the sound was recorded on a large distance range of the fiber.

### 3.2. Animals

**Figure 7:**
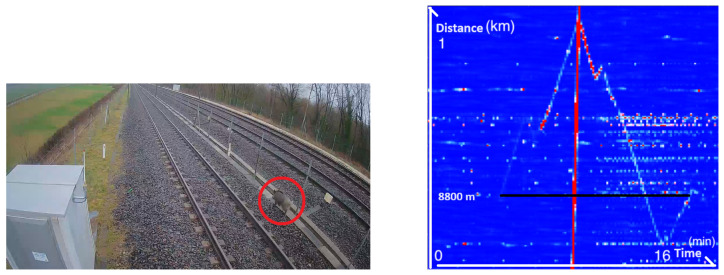
**Left**: Image taken from a CCTV camera showing the wild boar (red circle). **Right**: The associated frequency band energy between 0 and 250 Hz showing the boar trajectory.

Animal intrusion is a well-known problem in the rail industry. Some animals, such as wild boar, can cause collisions with trains, resulting in major delays to traffic. Acquisitions are carried out day and night, enabling us to capture all types of animal signals and visualize their trajectory. A wild boar was captured by the fiber in the middle of the day, as shown in Figure 7.

Note that the signal generated by the boar starts and stops at the same distance (around 8800 m), so we can assume that the boar enters and leaves the track at this point. This makes it possible to repair any damage inflicted on the fence and thus prevent any future wild boar intrusion at this location. The ends of the black line on the FBE plot show the boar’s entry and exit points. The start of the signal is weaker, suggesting that the boar was moving at some distance from the fiber. The noise can be explained by the rainy and windy weather that day. It should also be noted that a train passed by at the same time as the wild boar.

**Figure 8:**
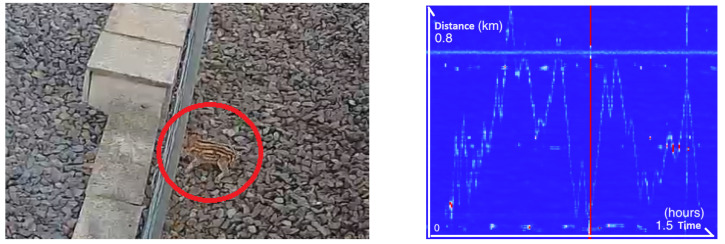
**Left**: Image taken from a CCTV camera showing the piglets (red circle). **Right**: The associated frequency band energy between 0 and 250 Hz showing the piglets trajectory.

The fiber was also able to detect wild boar piglets (Figure 8). There were a total of three piglets on the track. One of them traveled more than 3 km to find the exit, as it was trapped between the fence and the two tracks. The wild boar piglets’ entry and exit points are at around 6750 m. Note that the animals ran not on but beside the duct, which shows the great sensitivity of the sensing fiber.

**Figure 9:**
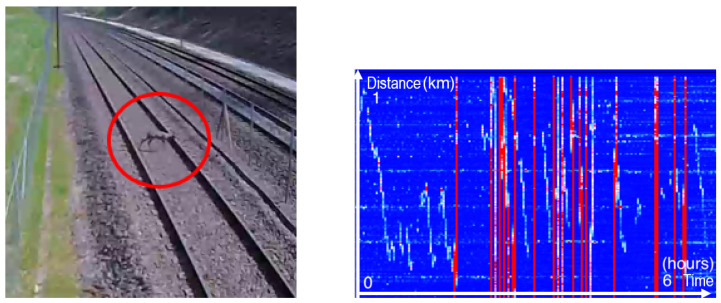
**Left**: Image taken from a CCTV camera showing the deer (red circle). **Right**: The associated frequency band energy between 0 and 250 Hz showing the deer trajectory.

The horizontal feature on the chart is caused by a constantly vibrating energy source near to the fiber, and this source can be some kind of beat harmonic between several sources of noise. At this point on the DTT, the cable is led away from the trackside gutter to an equipment cabinet where it is connected to the next section of the cable. A length of the cable is coiled near the cabinet as a service loop. This gives the appearance of an extended length of fiber detecting a vibration due to the physical layout of the cable. Other animals such as foxes, badgers, weasels, and even small rodents like mice were captured by the fiber. Finally, a deer got onto the railway line and traveled about 1 km over several hours, as shown in Figure 9.

All the vertical red lines represent the passage of various trains on the adjacent track. The other less intense signal is the deer’s trajectory.

### 3.3. Machine Learning

Based on the data acquired over time, FOSINA has been able to train a machine learning algorithm that can automatically classify detected signals and thus greatly improve intrusion monitoring on railway tracks.

#### 3.3.1. Dataset

**Figure 10:**
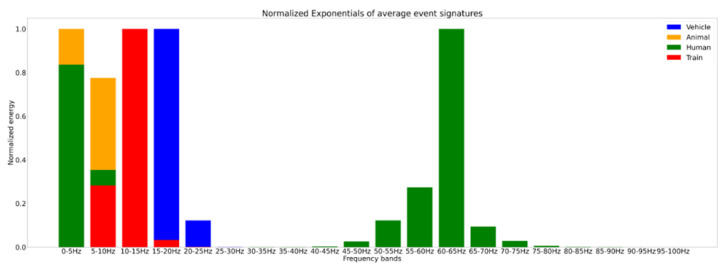
Average signals over the first 20 (0–100 Hz) FBE bands for different types of events (walking, train, animals, and car). We can see clear differences in the way the frequency content is split among the various bands.

This algorithm had been trained on data acquired at CEF over a year. Owing to CCTV access, multiple signals have been correctly labeled, in particular various animals (deers, boars, foxes, etc.) and vehicles (trucks, cars, excavators, etc.). The phase collected by the DxS has not undergone any pre-processing prior to the calculation of FBE. When looking at the various identified signals, patterns in the frequency content among similar signals have been noticed. When looking at the FBE bands for various signals, it was noticeable that all vehicle signals shared a similar distribution of energy; likewise, all walking signals also had a similar spread and so on. This constituted the intuition behind machine learning, which would automate this analysis. Figure 10 shows different detected events with their corresponding energy distribution with frequency.

Each signal of interest was thus extracted and labeled, resulting in a large dataset containing about 80 million samples, with each individual sample corresponding to one second of signal over 1.43 m. The dataset contains six classes: “No Signal”, “Human”, “Train”, “Vehicle”, “Animal”, and “Noise”. Among these, the “Noise” class contains all signals that are detected by the DxS but are not of interest to us.

**Figure 11:**
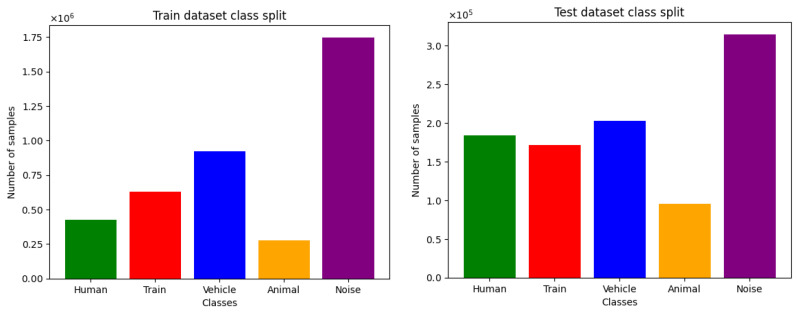
**Left**: Sample split among the five classes for the training set. **Right**: Sample split among the five classes for the testing set. In both cases, the “No Signal” class is not included.

The dataset is naturally imbalanced, with the “No Signal” class representing a large majority of samples (more than 90% of the dataset), which is due to the fact that, most of the time, there is no signal being detected on the fiber. The other five classes represent about five million samples altogether. For training and testing, the dataset is split into two parts as shown in Figure 11, with the training being further divided into a training and validation set.

From this, we can generate two types of datasets, one which uses the FBE bands as features, and one which uses the raw phase data as features. Each data point in the final dataset has 30 features. The first 21 of these features are the energy levels of FBE bands for 0–100 Hz, as that is where most of the frequency content is located. The last nine features are custom “distribution features”, which are computed directly from the histogram of FBE bands and give further information as to its distribution. As for the phase dataset, the features are the phase data itself and thus derive directly from the output data rate (ODR). Since the ODR for the CEF application was 500 Hz, we have 500 features for each data point.

#### 3.3.2. Model Architecture and Design

Multiple architectures and model types were experimented with. At first, we experimented with traditional ML models based on random forests (mainly extra random trees and gradient boosted trees). However, these proved to yield unsatisfying results when compared to neural network architectures, in particular one-dimensional CNNs (1DCNN). Random forests tended to result in accuracy scores averaging around 70% while being less computationally efficient than a simple 1DCNN. The use of CNNs was encouraged by the fact that they can automatically extract complex and hidden features from data, which proved useful to fully extract information from the FBE content of samples. One-dimensional CNNs have also proven successful when applied to DAS applications in the past, such as in Pipeline monitoring [21].

Another motivation for the use of a one-dimensional CNN was the need to keep the architecture relatively simple, with a lower number of parameters. This was in part due to the low dimensionality of our data (30 features per sample), but mainly due to the need for the model to perform in real-time applications, meaning that our model needed to be able to output predictions for an entire length of fiber every second. Thus, the model had to remain computationally light so as not to fall behind when working in real time.

Another approach that was experimented with was grouping the data into “tiles” of predetermined dimensions in terms of length and time. For example, one could arrange the data into tiles of 5 × 2 points, which would result in the model no longer processing individual data points representing one second in time by 1.43 m in space, but rather 5 s in time by 2.46 m in space. What this allows us to do first and foremost is to provide richer information to the model, as the data processed now includes information pertaining to the speed of the detected signal, which corresponds to its slope angle. But also, information regarding the signal’s width, which is in relation to the mass and length of the event generating the signal. Such tiling also has the side effect of allowing for more complex model architectures, since we both have a larger amount of information being fed into the model, but also less predictions to output at any given time. Indeed, proceeding from the previous example of a 5 × 2 tiling, this means that the model must output half the previous number of predictions, and now must do so every 5 s instead of every second as was the case previously. This approach provided a noticeable increase in performance when working with phase data, but that increase in performance was not replicated when working with FBE data, where the results were comparable to the previous fully real-time point-by-point approach. Thus, this method was retained when working with phase data as inputs but not with FBE.

In the end, this yielded two models: one fairly simple 1DCNN architecture, made to output predictions every second for the whole fiber, and one more complex architecture using a combination of time-distributed 1DCNN layers and a Bidirectional Long Short-Term Memory layer, made to output predictions on tiles of 5 by 5 points in the space and time dimensions, respectively. The choice of using a 5 by 5 tiling was made after testing various tile sizes, and it was found that a 5 by 5 window provided a healthy balance between adding enough spatial and time information while still retaining a large enough amount of samples for the model to train on (since the samples are tiled, the original size of the dataset is effectively divided by 25). When plugged into the model, the time distributed architecture trains five 1DCNNs in parallel, with each processing a “column” of the tiled data, i.e., five points in space by one second in time. Both models used the Adam optimizer with a default learning rate of 0.001. Both used the categorical cross entropy loss function, and both models were trained until the validation accuracy and loss values reached a consistent plateau. This was achieved in 10 and 16 epochs for the FBE and tiled phase model, respectively.

**Figure 12:**
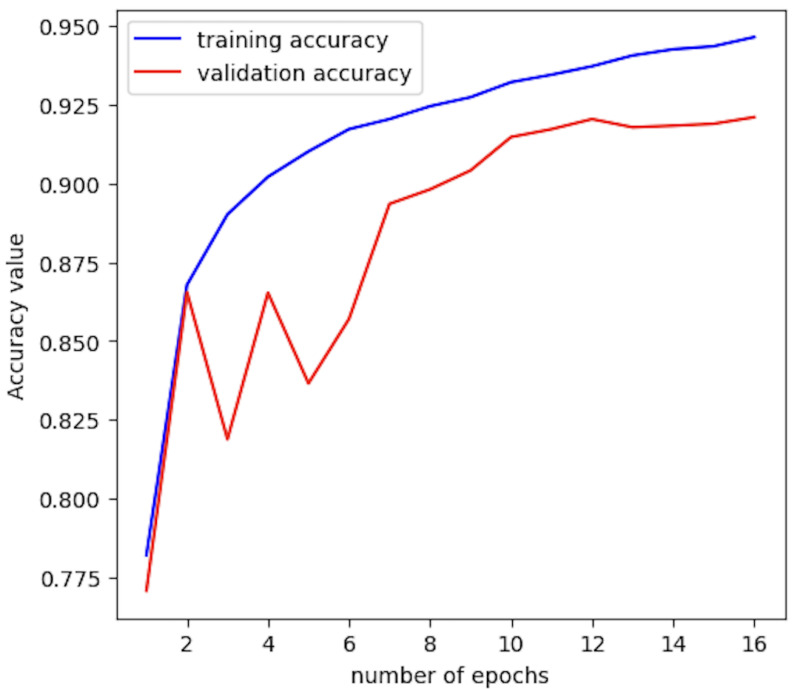
Accuracy evolution with iterations for the tiled phase model.

The final architectures for both the FBE and the tiled phase models are detailed in Table 2 and Table 3 respectively. The evolution of the model’s accuracy as a function of time for both validation and training is shown in Figure 12.

#### 3.3.3. Results

**Figure 13:**
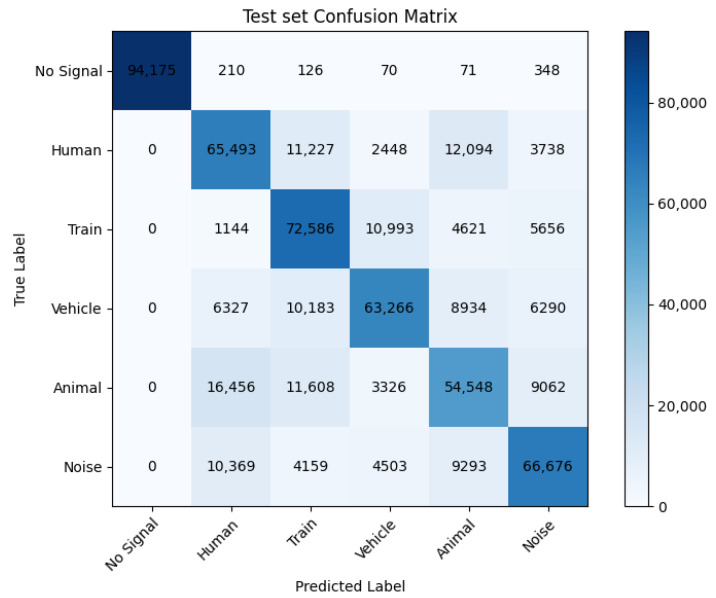
Confusion matrix for the FBE model.

**Figure 14:**
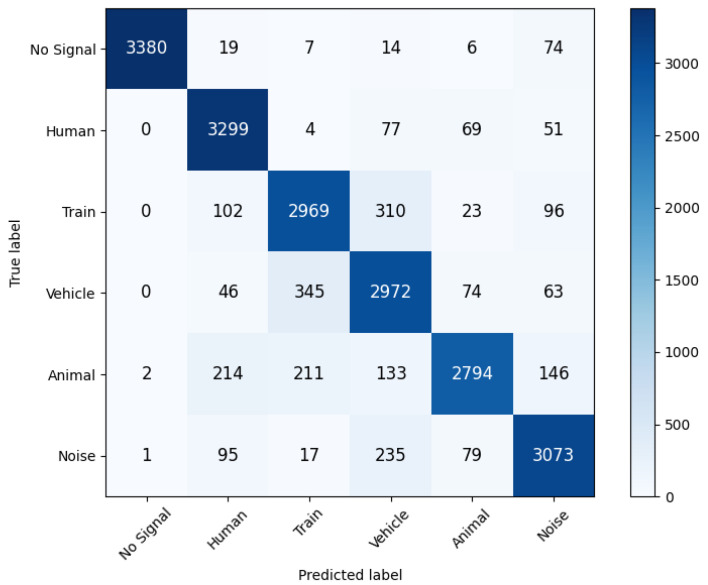
Confusion matrix for the tiled phase model.

Once trained, the model was evaluated on a balanced version of the testing dataset, resampled such that each class consisted of 95,000 samples in the case of the FBE dataset, and 3500 samples in the case of the tiled phase dataset. The precision/recall and f1 scores for each class, as well as the corresponding confusion matrix, are detailed in Table 4 and Figure 13 for the FBE model and in Table 5 and Figure 14 for the tiled phase model.

The final accuracy score for the FBE model is at about 73%. This might seem a rather low score, especially for certain classes, where it dips down to around 60%; however, this is often sufficient in practice. Indeed, this score is based on predictions made on individual samples, which are data points that span one second in time and 1.43 m in space. In any given signals, hundreds if not thousands of these data points are generated, such that, as long as we correctly identify a sufficient portion of them, the signal can be detected accurately.

**Figure 15:**
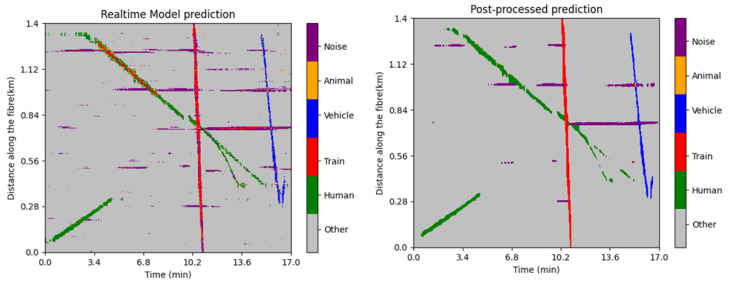
Model prediction on a patch of data containing various signals. **Left**: The real-time output of the model. **Right**: The post-processed prediction, where the misclassified portion of the walking signal has been corrected.

Additionally, the use of post-processing, with intuition as to what certain signals should look like, allows us to further refine predictions and correct eventual misclassifications. An example of such a case is illustrated in Figure 15, where in certain circumstances, a walking human would generate a signal much more similar to a train than a regular person. Using post-processing, we can correct such mistakes and prevent false positives.

The tiled phase model achieves much higher performance, reaching an accuracy score of 88%. This model mitigates or even eliminates some of the issues encountered with the FBE model, like the confusion between train and walking shown above. Another source of confusion for both models is that the edges of train signals can sometimes be misclassified as vehicle signals. This is due to the fact that the edges of train signals have a lower intensity value, as they represent the propagation of vibrations in front of and behind the train as it moves, and this lower intensity thus makes these signals more similar to vehicle signals. Once again, this can be addressed in post-processing by applying a smoothing filter to the prediction. Figure 15 also illustrates this, where we can see that on the raw prediction, the edges of the train signal are peppered with small misclassifications. Once the filter has been applied, however, these misclassifications are removed.

A further step in correcting misclassifications and handling false alarms is to apply some logic to alarm generation in the DxS software. The software version used in this study is v1.5.0-r24. For example, by applying a time threshold, we can make it such that a signal must last a minimum amount of time in order to generate an alarm. In practice, this allows us to eliminate a large majority of any false positives that might subsist after post-processing. False negative rate is harder to evaluate in practice, since we do not have permanent visibility over the whole track. We can gather a rough idea of it by comparing signals that have been picked up by the fiber but are classified as “No Signal” by the models. Such comparison shows that only very low intensity signals, generally characteristic of events happening further away from the fiber, can end up undetected by the models. This can be mitigated in practice by applying pre-processing methods to the data, such as filtering to emphasize certain frequencies. This, however, requires prior knowledge of the signals, in order to select the appropriate frequencies.

## 4. Discussion and Conclusions

### 4.1. Discussion

By combining DAS and 1D CNNs, our study offers a novel approach to detecting and classifying wildlife intrusion events, particularly those involving deer and wild boar, a topic that has received limited attention in prior research.

Some fiber has been deployed on the fence next to the railway lines. The data acquired with this configuration has shown some interesting signals, but more work is needed to present them clearly, particularly because of the greater exposure to environmental conditions such as rain and wind.

We are also aware that a study of the relationship between the amplitude of the signals picked up and the distance from the fiber would be interesting in order to gain a better understanding of the detection capabilities of the DxS.

Regarding the machine learning area, the use of FBE as features still has some advantages, mainly that of being agnostic with regards to sampling rate. The FBE model also allows for higher spatial and temporal precision. One intermediate solution would be to construct a model using phase data on the scale of individual samples. This approach has been experimented with as part of this study, but while it provided higher accuracy compared to the FBE model, it also drastically increased the computational cost, so much so that it made real time prediction untenable. Further pursuit of this approach would thus require further hardware or software optimization, as well as the use of acceleration techniques such as GPU acceleration. Another approach that could allow for higher time or space precision while using the tiled phase model is to apply it as a rolling window. However, this approach would still need to remain within the constraints of real time prediction. In the final analysis, it seems that, while tiled phase model represents the better option in most cases, FBE models could still prove useful in specific situations or as a steppingstone to provide intuition towards the construction of more complex models.

### 4.2. Conclusions

In this paper, we presented the results obtained by installing a fiber alongside a railway line. We showed that the DxS interrogator was capable of picking up a wide variety of signals, from the passage of a wild boar to the overflight of an aeroplane. The sensitivity of the fiber means that any activity occurring near railway tracks can be monitored in real time.

We also presented the performance and structure of our machine learning model, which can easily detect and classify different types of events such as the passage of a car, a train, a human, or even an animal in real time. Of the two models proposed in this paper, the tiled phase model seems the better option, owing to its higher performance.

Concerning the limitations of our study, it was carried out with a low sampling rate in order to be able to cover the entire system, which represents approximately 46 km. New acquisitions with a higher sampling rate and finer spatial resolution will make it possible to determine the various activity signatures in greater detail but over a shorter distance. In addition, the data acquired from the fiber attached to the fence could not be fully exploited because of the wet and windy weather during the acquisition campaign.

The purpose of this work was to introduce and present the capabilities of DxS in the railway sector. The next logical step is to determine the characteristics of the trains that will run on the track, such as speed and the number of bogies that make up the train. Finally, the identification of defects on railway tracks is another area of research for the coming years.

## Figures and Tables

**Table 1:** Acquisition parameters used for the tests.

Acquisition Parameters	
Sampling frequency (Hz)	500
Pulse duration (ns)	10
Gauge length (m)	5.7
Output spatial sampling (m)	1.43

**Table 2:** Final architecture for the model trained on FBE data.

Layer (Type)	Output Shape	Parameters Number
conv1d2 (Conv1D)	(None, 19, 64)	832
conv1d3 (Conv1D)	(None, 8, 64)	49,216
maxpooling1d1 (MaxPooling1D)	(None, 1, 64)	0
batchnormalization1 (BatchNormalization)	(None, 1, 64)	256
dropout1 (Dropout)	(None, 1, 64)	0
flatten1 (Flatten)	(None, 64)	0
dense3 (Dense)	(None, 64)	4160
dense4 (Dense)	(None, 32)	2080
dense5 (Dense)	(None, 6)	198
Total params: 56,742 (221.65 KB), Trainable params: 56,614 (221.15 KB)		

**Table 3:** Final architecture for the model trained on tiled phase data.

Layer (Type)	Output Shape	Parameters Number
timedistributed7	(None, 5, 2461, 64)	2624
timedistributed8	(None, 5, 123, 64)	0
timedistributed9	(None, 5, 123, 64)	256
timedistributed10	(None, 5, 123, 128)	163,968
timedistributed11	(None, 5, 12, 128)	0
timedistributed12	(None, 5, 12, 128)	512
timedistributed13	(None, 5, 1536)	0
bidirectional1	(None, 128)	819,712
dropout2	(None, 128)	0
dense6	(None, 64)	8256
dense7	(None, 32)	2080
dense8	(None, 6)	198
Total params: 997,606 (3.81 MB), Trainable params: 997,222 (3.80 MB)		

**Table 4:** Classification report for the FBE model on a balanced testing set. The numbered classes are in order 1: No Signal; 2: Human; 3: Train; 4: Vehicle; 5: Animal; 6: Noise.

Classification Report on Test Set				
**Class Number**	**Precision**	**Recall**	**f1-Score**	**Support**
1	1.00	0.99	1.00	95,000
2	0.65	0.69	0.67	95,000
3	0.66	0.76	0.71	95,000
4	0.75	0.67	0.70	95,000
5	0.61	0.57	0.59	95,000
6	0.73	0.70	0.71	95,000
accuracy			0.73	570,000
macro avg	0.73	0.73	0.73	570,000
weighted avg	0.73	0.73	0.73	570,000

**Table 5:** Classification report for the tiled phase model on a balanced testing set. The numbered classes are in order 1: No Signal; 2: Human; 3: Train; 4: Vehicle; 5: Animal; 6: Noise.

Classification Report on Test Set				
**Class Number**	**Precision**	**Recall**	**f1-Score**	**Support**
1	1.00	0.97	1.00	95,000
2	0.87	0.94	0.67	95,000
3	0.84	0.85	0.71	95,000
4	0.79	0.85	0.70	95,000
5	0.92	0.80	0.59	95,000
6	0.88	0.88	0.71	95,000
accuracy			0.88	21,000
macro avg	0.88	0.88	0.88	21,000
weighted avg	0.88	0.88	0.88	21,000

## Data Availability

Data are contained within the article.

## Abbreviations

The following abbreviations are used in this manuscript:

CEF	Centre d’Essais Ferroviaires
CCTV	Closed-Circuit TeleVision
CNN	Convolutional Neural Network
DAS	Distributed Acoustic Sensing
DFOS	Distributed Fiber Optic Sensing
DSS	Distributed Strain Sensing
DTGS	Distributed Temperature Gradient Sensing
DTS	Distributed Temperature Sensing
DTT	Dynamic Test Track
FBE	Frequency Band Energy
FBG	Fiber Bragg Grating
OFS	Optical Fiber Sensor
